# Navigating the Seas of Publications in A Medical Journal: The Role of an Editor

**DOI:** 10.7759/cureus.55233

**Published:** 2024-02-29

**Authors:** Shweta Tanwar

**Affiliations:** 1 Epidemiology and Public Health, Indian Council of Medical Research, New Delhi, IND

**Keywords:** research, scientific publication, publication ethics, biomedical journals, academic editor

## Abstract

The world of scientific publishing is a dynamic ecosystem where groundbreaking research and discoveries find their way into the public domain. Scientific journals play a pivotal role in disseminating new knowledge, shaping the healthcare landscape, and influencing clinical practice. Behind the scenes, editors serve as gatekeepers, meticulously reviewing and selecting articles to ensure the highest standards of quality and relevance. This article offers insights into the role of editors regarding publications in medical journals, shedding light on the challenges, responsibilities, and evolving trends in this crucial process.

## Editorial

Scientific journals stand as bastions of knowledge, disseminating innovative research to the global community. At the heart of this dissemination process lies the gatekeeping role of editors. Editors are the first line of defense in ensuring that the content published in scientific journals meets the highest standards of quality, credibility, and relevance. They are entrusted with the responsibility of sifting through a vast sea of submissions to identify those with the potential to contribute significantly to the field. They must ensure that the research presented is methodologically sound, ethically conducted, and adds value to the existing body of knowledge. As scientific publishing continues to evolve, the gatekeeping role of editors faces new challenges and opportunities. The rise of preprint servers, open science initiatives, and collaborative platforms introduces novel dynamics. Editors must remain agile, embracing innovations while upholding the core principles that safeguard the quality, integrity, and credibility of scientific knowledge.

Balancing innovation and rigor

One of the challenges faced by editors is striking the right balance between promoting innovation and the preservation of traditional scientific values. Striking this balance is vital to the evolution of scientific knowledge without compromising its foundational integrity. While journals seek to publish innovative research, they must also ensure that the methodologies employed are robust and the findings are reliable. On the other hand, editors should not always exclude studies with findings that are not statistically significant or that have inconclusive findings from consideration for publication. Such studies may provide evidence that, combined with that from other studies through meta-analysis, might still help answer important questions. Also, a public record of such negative or inconclusive findings may prevent unwarranted replication of effort or otherwise be valuable for other researchers considering similar work.

Addressing ethical concerns

Ethical considerations are paramount in the evaluation of biomedical publications. Editors act as guardians of ethical principles in scientific publishing. They establish and enforce guidelines that authors, reviewers, and other stakeholders must adhere to. By clearly communicating ethical expectations, editors create a framework that fosters responsible conduct and ensures the credibility of the published research. Editors must be vigilant in identifying potential ethical lapses, including plagiarism, data fabrication, and conflicts of interest. They use several guidelines as a reference for maintaining ethical standards, editorial independence, and transparency in their journals. The International Committee of Medical Journal Editors (ICMJE) serves as a guiding authority for editors by providing clear and standardized guidelines, promoting ethical practices, and supporting the integrity of the peer review and publication processes in the field of medical journals [1]. The Committee on Publication Ethics (COPE) provides valuable assistance to medical journal editors by offering ethical guidelines, educational resources, case consultation services, and a collaborative platform aimed at promoting ethical publishing practices [2,3]. Research involving human or animal subjects must adhere to ethical standards, and editors ensure that manuscripts comply with established guidelines, such as the Declaration of Helsinki for human research and the Animal Research: Reporting of In Vivo Experiments (ARRIVE) guidelines for animal studies [4,5]. They may seek additional information from authors, including documentation of ethical review board approvals to confirm the ethical conduct of the research.

Editors engage in continuous education to stay abreast of evolving ethical standards and guidelines. They foster ethical awareness among authors, reviewers, and fellow team members through training programs, workshops, and dissemination of best practices. Promoting a culture of ethical conduct contributes to the long-term integrity of the journal. Upholding ethical standards is not only a matter of maintaining the journal's reputation but also a crucial aspect of safeguarding the interests of patients and the broader scientific community.

The peer review process

Editors rely on the peer review process as a cornerstone for evaluating manuscripts. They carefully choose reviewers based on their knowledge, experience, and impartiality. Peer reviewers, who are experts in the field, assess the scientific merit, validity, and significance of the research. The editor's role involves selecting appropriate reviewers, overseeing the review process, and synthesizing reviewers' comments to provide constructive feedback to authors. The peer review process ensures a checks-and-balances system, allowing external experts to evaluate the manuscript's scientific rigor, clarity, and contribution to the field, thereby enhancing the overall quality of published research.

Adapting to technological advances

The landscape of scientific publishing is continually evolving, with technological advances reshaping the way research is conducted and disseminated. Editors must embrace innovations such as open-access publishing, preprint servers, and online submission systems. Adapting to these changes is crucial for improving accessibility, transparency, and information dissemination. For example, in scientific journals, with the advent and widespread usage of the internet and related technologies, the dissemination of scientific information has changed from physical printing to online print. Innovations in machine learning and artificial intelligence (AI) offer editors opportunities to enhance the peer review process [6]. Automated systems can assist in identifying potential reviewers, analyzing manuscript content, and even flagging statistical or methodological issues. While not a replacement for human judgment, AI can serve as a valuable complement, improving efficiency and accuracy. Technological tools facilitate enhanced collaboration and communication among editors, authors, and reviewers. Cloud-based platforms and communication tools enable real-time collaboration, reducing delays in decision-making and fostering a more transparent and interconnected editorial workflow.

Online platforms break down geographical barriers, allowing researchers from diverse regions to contribute and access knowledge. Adapting to technological advances ensures that scientific publications are accessible on a global scale. This global reach enhances the impact and influence of scientific publications. As technology continues to advance, editors must remain agile, integrating new tools and approaches to navigate the evolving landscape of scientific publishing. In doing so, editors not only contribute to the progress of their respective journals but also play a pivotal role in shaping the future of scholarly communication.

Global perspectives and diversity

Scientific research often involves diverse cultural contexts, and editors must navigate potential ethical sensitivities associated with cultural or regional differences. They play a pivotal role in promoting diversity and inclusivity in medical research. Ensuring a variety of perspectives, methodologies, and geographical representations enriches the scientific discourse. Editors must be mindful of biases in the selection process and actively seek to amplify voices that may have been historically underrepresented in medical literature. They also strive to ensure that research involving vulnerable populations is conducted ethically and that studies respect the cultural nuances and norms of the communities involved.

Collaboration with authors

Successful publication involves a collaborative effort between authors and editors. Effective communication, constructive feedback, and transparent editorial processes contribute to a positive author-editor relationship. Editors should provide authors with clear guidelines, address queries promptly, and offer guidance on how to improve their submissions. Maintaining a transparent and collaborative relationship with authors is essential for fostering a positive and productive publishing environment.

In conclusion, being an editor of a scientific journal is a multifaceted role that involves juggling scientific expertise, ethical acumen, and effective communication skills. The decisions made by editors shape the narrative of medical research, influence clinical practices, and contribute to the advancement of healthcare. Editors, with their commitment and insight, significantly influence the direction of scientific progress. They ensure that the vast sea of scientific submissions in their journal is navigated with precision and purpose, and the gates they maintain serve as a guiding light for rigor and relevance in the continually expanding realm of knowledge.

## References

[1] International Committee for Medical Journal Editors. (2023). Up-dated ICMJE recommendations | International Committee for Medical Journal Editors. https://icmje.org/news-and-editorials/updated_recommendations_may2023.html.

[2] (2023). Ethics toolkit for a successful editorial office | Committee on Publication Ethics. https://publicationethics.org/resources/guidelines/ethics-toolkit-editors.

[3] (2023). Principles of transparency and best practice in scholarly publishing | Committee on Publication Ethics. https://publicationethics.org/resources/guidelines/principles-transparency-and-best-practice-scholarly-publishing.

[4] World Medical Association (2013). World Medical Association Declaration of Helsinki: ethical principles for medical research involving human subjects. JAMA.

[5] Percie du Sert N, Hurst V, Ahluwalia A (2020). The ARRIVE guidelines 2.0: Updated guidelines for reporting animal research. PLoS Biol.

[6] Checco A, Bracciale L, Loreti P (2021). AI-assisted peer review. Humanit Soc Sci Commun.

